# Most Promising Emerging Therapies for Pulmonary Fibrosis: Targeting Novel Pathways

**DOI:** 10.3390/biomedicines14010154

**Published:** 2026-01-11

**Authors:** Lorenzo Carriera, Roberto Lipsi, Meridiana Dodaj, Riccardo Inchingolo, Andrea Smargiassi, Angelo Coppola, Pier-Valerio Mari, Roberto Barone, Simone Ielo, Raffaele Scala, Luca Richeldi

**Affiliations:** 1 Facoltà di Medicina e Chirurgia, Università Cattolica del Sacro Cuore, 00168 Rome, Italy; roberto.barone01@icatt.it; 2 Department of Pulmonology and Sub-Intensive Respiratory Unit, Ospedale Santa Maria della Misericordia, 06156 Perugia, Italy; roberto.lipsi@ospedale.perugia.it (R.L.); meridiana.dodaj@ospedale.perugia.it (M.D.); 3 UOC Pneumologia, Dipartimento Neuroscienze, Organi di Senso e Torace, Fondazione Policlinico Universitario A. Gemelli IRCCS, 00168 Rome, Italy; riccardo.inchingolo@policlinicogemelli.it (R.I.); andrea.smargiassi@policlinicogemelli.it (A.S.); luca.richeldi@policlinicogemelli.it (L.R.); 4 UOC Pneumologia, Ospedale San Filippo Neri—ASL Roma 1, 00135 Rome, Italy; angelo.coppola@aslroma1.it; 5 Internal Medicine, San Carlo di Nancy Hospital, 00165 Rome, Italy; piervalerio.mari@gmail.com; 6 Pulmonology and Respiratory Intensive Care Unit, Ospedale San Donato, USL Toscana Sud-Est, 52100 Arezzo, Italy; ielosimone1@gmail.com (S.I.); raffaele.scala@uslsudest.toscana.it (R.S.)

**Keywords:** pulmonary fibrosis, IPF, PPF, antifibrotic therapy, nerandomilast

## Abstract

Interstitial lung diseases (ILDs) encompass a heterogeneous group of disorders characterized by varying degrees of inflammation and fibrosis. Despite advances in understanding the pathogenesis, therapeutic options remain limited, particularly for patients with progressive phenotypes. Current international guidelines for idiopathic pulmonary fibrosis (IPF) and progressive pulmonary fibrosis (PPF) emphasize the need for antifibrotic strategies and call for novel pharmacological interventions targeting key molecular pathways involved in fibrogenesis. This review provides a comprehensive overview of the most promising emerging pharmacological agents for ILDs, with particular attention to their mechanisms of action, efficacy, and safety profiles as reported in recent preclinical and clinical studies. The recent approval of Nerandomilast and the ongoing phase III trials of other agents mark a pivotal transition toward a new generation of antifibrotic therapies, aiming to achieve more effective disease control and improved patient outcomes. In view of an enlargement of active drugs aiming at controlling the disease with different mechanisms, the Authors underline the need for a “precision medicine” model to be applied to each ILD phenotyped patient, mirroring what already happens for other respiratory diseases.

## 1. Introduction

Pulmonary fibrosis represents a heterogeneous group of chronic respiratory diseases characterized by abnormal accumulation of fibrotic tissue leading to thickening and stiffening of the lung parenchyma. This pathological remodeling results in reduced lung compliance, impaired gas exchange, and progressive dyspnea, ultimately culminating in irreversible structural damage and respiratory failure [1].

Idiopathic pulmonary fibrosis (IPF) is the most common type of fibrosing interstitial lung disease worldwide [2]. It predominantly affects older adults and is characterized by progressive dyspnea, declining lung function, and a poor prognosis.

The pathogenesis of pulmonary fibrosis is complex and has not yet been fully elucidated. Multiple risk factors converge to initiate and perpetuate this process. Occupational and environmental exposures (e.g., asbestos, silica, organic antigens) can directly damage the lung and promote chronic inflammatory–fibrotic signaling [3]. Lifestyle factors such as smoking further amplify oxidative stress and epithelial injury, while comorbidities including gastroesophageal reflux and chronic viral infections may sustain microinjury and inflammation [4]. Superimposed on these external drivers, genetic susceptibility affecting epithelial integrity, telomere maintenance, and host defense mechanisms increase vulnerability of the alveolar epithelium [5,6,7]. In response to injury, activated epithelial cells release a broad array of profibrotic mediators, including transforming growth factor-β (TGF-β), platelet-derived growth factor, connective tissue growth factor, and other cytokines and chemokines [3]. These signals promote fibroblast recruitment, proliferation, and differentiation into myofibroblasts, the principal effector cells responsible for extracellular matrix (ECM) production [8]. Myofibroblasts secrete excessive amounts of collagen and other matrix components, leading to progressive matrix accumulation and increased tissue stiffness. ECM remodeling is not merely a consequence of fibrosis but an active driver of disease progression [9]. Matrix stiffening alters mechanotransduction signaling [10], further activating fibroblasts and reinforcing profibrotic pathways such as TGF-β, Wnt/β-catenin, Hedgehog, Rho–ROCK, and integrin-mediated signaling. In parallel, immune dysregulation and chronic low-grade inflammation contribute to disease amplification through macrophage polarization, cytokine release, and crosstalk with mesenchymal and epithelial compartments [11]. Mechanistically, pulmonary fibrosis is driven by the activation of several interconnected signaling pathways. Transforming growth factor-β (TGF-β)/Smad signaling represents a central profibrotic axis, promoting fibroblast activation, myofibroblast differentiation, and ECM production [12,13]. This pathway interacts with other intracellular cascades, including MAPK and PI3K/AKT/mTOR signaling, which support cell proliferation, survival, and metabolic adaptation within fibrotic tissue [14]. In parallel, developmental pathways that are normally active during lung development and repair, such as Wnt/β-catenin, Notch, and Hippo/YAP-TAZ, become aberrantly reactivated in disease [15]. Their persistent activation disrupts normal epithelial regeneration, sustains fibroblast survival, and contributes to the maintenance of a profibrotic tissue environment [15]. Additional mechanisms further amplify disease progression. Oxidative stress, largely driven by mitochondrial dysfunction and NADPH oxidase activity, enhances epithelial injury and strengthens profibrotic signaling [16]. At the same time, impaired regulation of autophagy and apoptosis leads to the accumulation of damaged or senescent cells, preventing effective tissue repair [17]. Finally, epigenetic alterations, including changes in DNA methylation, histone modifications, and RNA-based regulation, stabilize profibrotic gene expression programs across epithelial, immune, and mesenchymal cells, helping to explain the chronic and self-perpetuating nature of fibrosis despite diverse initiating insults [18]. Finally, emerging evidence implicates the microbiome, both lung and gut, in modulating immunity and systemic inflammation through the gut–lung axis [19,20]. Ultimately, the failure of effective epithelial regeneration, depletion or dysfunction of resident progenitor cells, persistent myofibroblast activation, and self-perpetuating matrix remodeling create a feed-forward loop that sustains fibrogenesis independently of the initial injurious trigger.

In recent years, the concept of Progressive Pulmonary Fibrosis (PPF) has gained prominence in the scientific community, shifting the therapeutic focus from a nosological classification to one based on the clinical behaviour of the disease: progressive phenotype. In fact, despite arising from different etiologies, PPF shares a similar pattern of disease progression with IPF [1]. This has opened the way for shared therapeutic strategies and greater attention to early diagnosis [21].

Current guidelines on IPF and PPF recommend the use of two antifibrotics: pirfenidone and nintedanib in IPF, and nintedanib with a conditional recommendation in PPF [1]. Both these drugs represented a significant advance in the treatment of pulmonary fibrosis, being able to slow down the progression of the disease and improve disease outcomes (Table 1).

Pirfenidone is a low molecular weight compound with antifibrotic, anti-inflammatory, and antioxidant activity. Its exact mechanism is not fully understood, but it is believed to inhibit the synthesis of TGF-β and other fibrogenic mediators [22].

**Table 1:** Approved antifibrotic agents for IPF and PPF.

Drug	Company	Mechanism/Target Pathway	Clinical Trial	Main Findings	Year of Approval	Approved Indication
Pirfenidone	Roche	Antifibrotic, anti-inflammatory, and antioxidant agent; inhibits synthesis of TGF-β and other fibrogenic mediators (exact mechanism not fully understood)	CAPACITY and ASCEND trials	Significantly reduced the decline in FVC compared with placebo in IPF patients [23,24]	2008 Japan, 2011 EMA, 2014 FDA	IPF
Nintedanib	Boehringer Ingelheim	Oral tyrosine kinase inhibitor targeting VEGF, FGF, and PDGF receptors involved in pulmonary fibrogenesis	INPULSIS and INBUILD trials	INPULSIS: significant reduction in annual rate FVC decline in IPF patients [25]; INBUILD: 57% reduction in annual FVC decline in non-IPF progressive fibrosing ILD compared to placebo [26]	2014 FDA, 2015 EMA	IPF and PPF

The table outlines each drug’s mechanism of action or targeted pathway, the pivotal clinical trials that supported regulatory approval, the main efficacy and safety findings from these trials, and the current clinical indications for each therapy.

It is approved in IPF based on the CAPACITY [23] and ASCEND [24] trials who demonstrated a significant reduction in the decline in forced vital capacity (FVC) in patients treated with pirfenidone compared to placebo. The most frequent side effects are: photosensitivity, nausea, dyspepsia, increased liver enzymes.

Nintedanib is an oral tyrosine kinase inhibitor that targets multiple growth factors receptors, including VEGF, FGF, and PDGF, all of which are involved in pulmonary fibrogenesis. The INPULSIS trial [25] showed a significant reduction in FVC decline in IPF patients treated with nintedanib. Later, the INBUILD trial [26], evaluating the efficacy of nintedanib in patients with non-IPF progressive fibrosing ILDs, demonstrated a 57% reduction in the annual rate of decline in FVC compared to placebo. Therefore, Nintedanib was approved for the treatment of chronic fibrosing ILD with a progressive phenotype (PPF) by the major regulatory agencies (EMA, FDA).

Its main side effects are diarrhoea (very common), nausea, hepatotoxicity, and an increased risk of cardiovascular events, especially in older subjects [27].

Despite the progress made with the introduction of these drugs, pirfenidone and nintedanib, are not curative: they slow the progression of fibrosis but do not block or reverse it. Furthermore, some patients show poor clinical response or reduced tolerability. Consequently, scientific research has focused efforts on the development of new molecules, combination therapies and personalized pharmacological approaches.

However, the complexity and heterogeneity of pulmonary fibrosis continue to pose major challenges: variability in disease behavior, delayed diagnosis, limited biomarkers for treatment response, and difficulties in designing robust clinical trials all hinder the path to new approvals. One of the main limitations in advancing personalized approaches in IPF and other progressive fibrosing ILDs is the persistent lack of validated biomarkers capable of predicting treatment response. Extensive research has identified multiple circulating biomarkers associated with epithelial injury, ECM remodelling, inflammation, and fibrosis, including surfactant proteins A and D (SP-A, SP-D), matrix metalloproteinases (particularly MMP-7), Krebs von den Lungen-6 (KL-6), S100A12, procollagen III N-terminal peptide (PIIINP), galectin-3, periostin, and dysregulated microRNAs [28]. However, none has been validated to reliably predict response to antifibrotic therapy or guide treatment selection in routine clinical practice. Another unmet need concerns the identification of endpoints beyond lung function decline, including patient-centered outcomes such as clinical, physiological, and radiological assessments and quality of life, as well as therapeutic strategies to act against the life-threatening acute exacerbations of the underlying ILD [29].

This review provides an overview of recent advances in pharmacological research for the management of ILDs, focusing on the most relevant clinical trials and their potential implications for therapeutic strategies (Table in Section 9). Notably, more than 100 pharmacological agents are currently being evaluated in this field, highlighting the breadth and dynamism of ongoing research [30]. The mechanisms of action of the most promising emerging drugs across all study phases are discussed in detail, and a dedicated section summarizes additional pathogenic pathways and candidate molecules currently under investigation, particularly those in early-phase clinical development.

## 2. PDE4B Inhibition

Cyclic adenosine monophosphate (cAMP) is a ubiquitous intracellular second messenger that plays a critical role in the function of different organs, including the lung, the CNS, inflammation and immunity, and the endocrine system. Cellular cAMP levels are tightly regulated by phosphodiesterases, particularly the cAMP-specific phosphodiesterase-4 (PDE4) family, which selectively hydrolyzes cAMP and thereby modulates the activity of downstream effectors such as protein kinase A (PKA), exchange proteins directly activated by cAMP (EPAC), and Popeye domain–containing proteins [31]. Through these mechanisms, PDE4 enzymes act as key regulators of inflammatory and fibrotic signaling pathways [32]. Targeting PDE4 has therefore emerged as a rational therapeutic strategy in obstructive and fibrotic lung diseases, providing the mechanistic foundation for the development of novel agents such as Nerandomilast.

Nerandomilast is an oral preferential phosphodiesterase-4B (PDE4B) inhibitor. By inhibiting PDE4B, Nerandomilast increases intracellular cAMP concentrations, resulting in the modulation of multiple inflammatory and profibrotic signaling pathways [33].

This also leads to decreased production of pro-inflammatory cytokines such as tumor necrosis factor-alpha (TNF-α), interleukin-6 (IL-6), and transforming growth factor-beta (TGF-β) [34], as well as suppression of fibroblast proliferation, differentiation into myofibroblasts, and ECM deposition [35,36]. It enhances the action of antifibrotic mediators such as prostaglandin E2 (PGE2), prostacyclin and adenosine, blocking fibroblast activation, making fibroblasts more prone to apoptosis, and preserving the integrity of alveolar epithelial cells [31].

Additionally, PDE4B inhibition exerts immunomodulatory effects by dampening neutrophilic and macrophagic inflammatory responses [37], which are implicated in the pathogenesis and progression of fibrotic lung disease [38].

**Figure 1:**
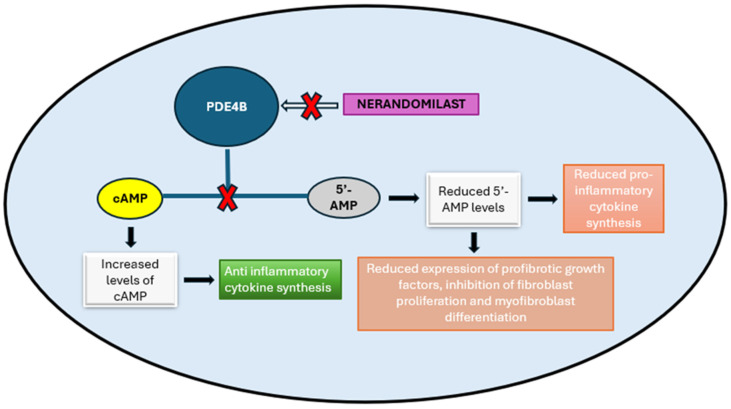
Nerandomilast inhibits PDE4B activity and modulates downstream fibrotic and inflammatory pathways.

A schematic representation of the mechanism of action of Nerandomilast is presented in Figure 1.

Recent phase III clinical trials, FIBRONEER-IPF [39] and FIBRONEER-ILD [40], have provided robust evidence supporting the efficacy and safety of Nerandomilast in IPF and PPF populations.

In the FIBRONEER-IPF trial, which enrolled 1177 patients with IPF, Nerandomilast significantly reduced the decline in FVC compared to placebo. Patients treated with 18 mg twice daily exhibited an adjusted mean difference in FVC decline of 68.8 mL (*p* < 0.001), while the 9 mg twice daily group showed a reduction of 44.9 mL (*p* = 0.02) [39].

Similarly, the FIBRONEER-ILD trial, involving 1176 patients with PPF, demonstrated that Nerandomilast significantly slowed the decline in FVC versus placebo. The adjusted mean change in the FVC at week 52 was −98.6 mL (95% confidence interval [CI], −123.7 to −73.4) in the Nerandomilast 18 mg group, −84.6 mL (95% CI, −109.6 to −59.7) in the Nerandomilast 9 mg group, and −165.8 mL (95% CI, −190.5 to −141.0) in the placebo group [40]. Additionally, there was a trend toward reduced mortality risk in patients treated with Nerandomilast 18 mg (hazard ratio 0.77, *p* = 0.06), although this did not reach statistical significance [40]. Despite not meeting secondary endpoints (risk of acute exacerbation, hospitalization for respiratory cause, or death), a pooled analysis of the FIBRONEER trials showed that Nerandomilast 18 mg was associated with a nominally significant reduction in the risk of death by 43% overall, by 59% in patients without background therapy, and by 41% in patients with background Nintedanib [41].

Regarding safety, the adverse event profile of Nerandomilast in both FIBRONEER studies was consistent with previous phases. The most commonly reported adverse event was diarrhoea, occurring in 17.5% of patients receiving 9 mg and 27.4% receiving 18 mg, compared to 14.6% in the placebo group. Serious adverse event rates were comparable across treatment arms, supporting a favourable safety and tolerability profile.

On the basis of the results of FIBRONEER trials, Nerandomilast has been recently approved by FDA for the treatment of IPF, being the first new treatment option in over a decade [42].

An open-label extension trial of the long-term safety and efficacy of Nerandomilast in IPF and PPF is currently recruiting patients who completed those trials (FIBRONEER-ON, NCT06238622) [43].

## 3. TGF-β Inhibition with Inhaled Pirfenidone and Structural Analogues

To overcome the limitations due to oral pirfenidone which can lead to dose reductions or treatment discontinuation, an inhaled formulation of pirfenidone (AP01) has been developed to deliver the drug directly to the lungs, maximizing the local antifibrotic effects while minimizing systemic exposure.

The most advanced clinical evaluation of inhaled pirfenidone comes from a randomized, double-blind, dose-ranging phase Ib study [44], which enrolled 91 patients with IPF who were either unable to tolerate or ineligible for oral antifibrotics. Participants were randomized to receive nebulized AP01 at either 50 mg once daily (QD) or 100 mg twice daily (BID), administered via a handheld nebulizer over a treatment period of up to 72 weeks. At 48 weeks, the higher-dose group (100 mg BID) exhibited remarkable stability in lung function, with a mean decline in percent predicted FVC of only −0.4% (approximately −34 mL), compared to a −4.9% decline (around −188 mL) in the 50 mg QD group. In addition to the preservation of lung function, inhaled pirfenidone demonstrated a favourable safety and tolerability profile. The incidence of systemic side effects commonly seen with oral pirfenidone—such as nausea, dyspepsia, weight loss, and rash—was markedly reduced. Adverse events were predominantly mild, with the most frequent being cough and throat irritation related to the inhalation route. Importantly, the rate of treatment discontinuation due to adverse events was low, indicating that inhaled administration may improve long-term adherence. Long-term safety and efficacy have also been explored in the AP01-005 extension study, which included both IPF and non-IPF fibrosing ILD patients. In this open-label follow-up, a subset of participants remained on inhaled pirfenidone for up to 240 weeks. These patients maintained stable FVC trajectories without evidence of cumulative toxicity. Furthermore, pharmacokinetic data from early-phase trials confirmed that inhaled delivery results in significantly lower systemic drug levels compared to oral dosing, while achieving high local concentrations in the pulmonary parenchyma, where the antifibrotic action is needed most [45].

The inhaled route for this drug may allow also for synergistic or combination strategies with other agents, leading to reduced systemic burden and drug–drug interaction potential, this being particularly important in patients with limited tolerance or receiving concurrent systemic therapies [46].

A randomized, double-blind, placebo-controlled phase 2b clinical study (NCT06329401) [47] is currently being conducted in order to assess the efficacy of two doses of inhaled pirfenidone versus placebo in addition to standard of care in patients with PPF over 52 weeks. Furthermore, two structural analogues of pirfenidone, SC101 (sufenidone) and HEC585 (yfenidone) are under investigation in phase 2/3 trials (NCT06125327) [48] in IPF and phase 2 RCTs (NCT05060822) [49] in IPF and PPF (NCT05139719) [50].

## 4. Lysophosphatidic Acid Pathway

Lysophosphatidic acid (LPA) is a bioactive phospholipid that functions as an extracellular signaling molecule through a family of G protein-coupled receptors (LPA_1_–LPA_6_). The LPA signaling axis is involved in a variety of physiological and pathological processes, including cell migration, proliferation, survival, angiogenesis, and, notably, fibrosis [51]. In IPF, LPA is primarily generated by the enzyme autotaxin (ATX), which converts lysophosphatidylcholine (LPC) into LPA. Once produced, LPA binds to LPA_1_ receptors expressed on epithelial cells, endothelial cells, and fibroblasts, triggering a cascade of pro-fibrotic responses, such as increased vascular permeability, recruitment and activation of fibroblasts, differentiation into myofibroblasts, ECM deposition, including collagen, and apoptosis of alveolar epithelial cells [52].

BMS 986020 is first generation oral selective LPA_1_ receptor antagonist for the treatment of IPF. In a Phase 2 clinical trial (NCT01766817), the drug demonstrated efficacy in slowing lung function decline: patients receiving the higher dose (600 mg twice daily) showed a statistically significant reduction in the rate of decline in FVC compared to placebo over a 26-week period [53].

Despite these promising results, the development of BMS 986020 was discontinued due to safety concerns. Specifically, patients experienced hepatobiliary adverse events, including elevated liver enzymes, cholecystitis, mechanistic findings in preclinical models indicating inhibition of bile acid transporters (e.g., BSEP, MRP3/4) and mitochondrial toxicity in cholangiocytes [54].

Following the discontinuation of BMS 986020, attention turned to second-generation LPA_1_ antagonists with improved safety profiles. The most notable of these is Admilparant (BMS 986278), which retains selective LPA_1_ inhibition but was structurally optimized to reduce hepatobiliary toxicity. Admilparant in a phase 2 double-blind RCT (NCT04308681) conducted on IPF and PPF patients demonstrated efficacy by reducing FVC changes over 26 weeks compared to placebo, with an acceptable safety and tolerability profile. The rate of change in predicted FVC in IPF was −2.7% for placebo vs. −2.8% and −1.2% for the 30 mg and 60 mg doses, respectively [55]. A more pronounced benefit was seen in PPF cohort, with the decline in FVC of −4.3%, −2.7%, and −1.1% in the placebo, 30 mg, and 60 mg groups, respectively. Moreover, a post hoc analysis showed that treatment with 60 mg Admilparant delayed time to disease progression over 26 weeks compared with placebo in both the IPF (HR, 0.54; 95% CI, 0.31–0.95) and PPF (HR, 0.41; 95% CI, 0.18–0.90) cohorts [56].

Currently, the efficacy of BMS-986278 is under further evaluation in IPF and PPF in phase 3 RCTs, NCT06003426 for IPF [57] and NCT06025578 for PPF [58].

## 5. Prostacyclin–cAMP Signaling

Prostacyclin signaling has emerged as an important regulator of vascular homeostasis, inflammation, and tissue remodeling, processes that are closely intertwined in pulmonary fibrosis. Prostacyclins exert their biological effects primarily through activation of the prostacyclin (IP) receptor, which stimulates adenylate cyclase and increases intracellular cAMP levels [59,60].

cAMP-dependent signaling has been shown to modulate fibrocyte adhesion, differentiation, and proliferation, partly through suppression of extracellular signal-regulated kinase 1/2 (ERK1/2) within the mitogen-activated protein kinase (MAPK) pathway. In addition, prostacyclin signaling can interfere with profibrotic pathways by modulating Smad-dependent signaling, suggesting functional crosstalk with transforming growth factor-β (TGF-β) [60]. Beyond its effects on cellular behavior, cAMP signaling also influences ECM remodeling by activating transcriptional programs mediated by cAMP response element–binding protein (CREB), thereby limiting de novo deposition of collagen type I, collagen type III, and fibronectin [60].

Within this mechanistic framework, Treprostinil, a prostacyclin analogue approved for the treatment of pulmonary arterial hypertension (PAH) [61], has been investigated for its potential antifibrotic effects. By activating adenylate cyclase to produce cAMP and increasing intracellular cAMP levels, Treprostinil attenuates TGF-β–driven profibrotic signaling and ECM production, providing a biological rationale for its evaluation in fibrotic lung disease [62].

The INCREASE trial (NCT02630316) [63] was a phase III RCT investigating efficacy and safety of Treprostinil treatment in PAH due to ILDs. It significantly improved exercise tolerance measured with 6 min walking test at 16 weeks (+31 m vs. placebo; *p* < 0.001), along with favourable trends in NT-proBNP levels and time to clinical worsening. These benefits were consistent across various ILD subtypes, including IPF, connective tissue disease-associated ILD, and combined pulmonary fibrosis and emphysema (CPFE). Importantly, a post hoc analysis from the INCREASE trial also suggested potential antifibrotic effects of Treprostinil, showing a slower decline in FVC over time [64].

Building on the positive outcomes of the INCREASE trial, the TETON program was initiated to further explore the effects of inhaled Treprostinil in patients IPF and PPF, even in the absence of pulmonary hypertension [65].

The TETON trials, two phase III RCTs, NCT04708782 [66] and NCT05255991 [67], evaluating the efficacy of inhaled Treprostinil in treating IPF, are currently underway. Together, they are enrolling over 1100 patients with confirmed IPF. These studies are evaluating whether inhaled Treprostinil, administered via nebulizer four times daily, can slow disease progression as measured by change in FVC over 52 weeks. Secondary endpoints include time to clinical worsening, time to first acute exacerbation of IPF, overall survival [65]. A third study, TETON-PPF [68], is focusing specifically on patients with non-IPF progressive pulmonary fibrosis, a population with similarly poor prognosis and limited treatment options.

## 6. Angiotensin Type 2 Receptor Signaling

Dysregulation of the renin–angiotensin system has been implicated in the pathogenesis of idiopathic pulmonary fibrosis, particularly through its effects on alveolar epithelial injury and fibroblast activation. In fibrotic lungs, apoptotic alveolar epithelial cells release angiotensinogen, which is converted to angiotensin II and predominantly signals through the angiotensin type 1 receptor (AT1R), promoting epithelial cell apoptosis and stimulating fibroblast-driven collagen production. In contrast, activation of the angiotensin type 2 receptor (AT2R) is associated with anti-inflammatory, anti-apoptotic, and antifibrotic effects, and is increasingly recognized as part of a protective, counter-regulatory pathway within the angiotensin system [69].

Experimental activation of AT2R has been shown to attenuate bleomycin-induced pulmonary fibrosis, supporting a role for this pathway in limiting fibrotic remodeling [70]. AT2R signaling promotes alveolar epithelial repair by supporting the function of type 2 alveolar epithelial cells (AEC2), enhancing surfactant production, preserving alveolar integrity, and facilitating regeneration of type 1 epithelial cells (AEC1), which are typically compromised in IPF. These effects are accompanied by reduced fibroblast activation, collagen deposition, and overall fibrotic burden [70].

Buloxibutid (C21), an oral angiotensin type 2 receptor (AT2R) agonist, recently underwent a phase 2, multicentre, open-label, 36 weeks, single-armed trial, called AIR, NCT04533022, evaluating its efficacy and safety in patients with IPF [71]. The study enrolled 52 patients with confirmed IPF who received oral Buloxibutid at 100 mg twice daily for 24 weeks, with an optional extension to 36 weeks. FVC remained stable at 24 weeks and improved at 36 weeks, together with an observed reduction in plasma profibrotic cytokine TGF-β1 and increase in collagenase MMP-13 levels. Treatment was well tolerated, with no serious drug-related adverse events [72].

The ASPIRE study (NCT06588686) is a global, randomized, double-blind, placebo-controlled, 52-week phase 2b clinical trial evaluating the efficacy and safety of Buloxibutid in patients with IPF. Participants are randomized to receive Buloxibutid (100 mg or 50 mg twice daily) or placebo, in addition to standard therapy with Nintedanib. The primary endpoint is change from baseline in FVC. The study includes 270 patients from 14 countries. It is currently recruiting patients and results of this study are greatly anticipated [73].

## 7. ROCK2 Inhibition

ROCK2 is a serine/threonine kinase belonging to the Rho-associated coiled-coil-containing protein kinase (ROCK) family, which includes two main isoforms: ROCK1 (localized primarily in the heart, lungs, and hematopoietic cells), ROCK2 (expressed primarily in the brain, lungs, liver, and smooth muscle tissue) [74].

ROCK2 has specific roles in fibrotic, immune, and inflammatory processes [75]. Activation of this pathway promotes the release of profibrotic and proinflammatory mediators, including tumor necrosis factor-α, transforming growth factor-β1 (TGF-β1), and interleukin-4, and influences immune cell polarization within the fibrotic lung microenvironment. In addition, Rho–ROCK signaling is closely linked to oxidative stress, a key contributor to fibrotic lung injury, with experimental evidence showing that ROCK inhibition attenuates oxidative damage in hypoxia-induced lung injury models [76].

Its inhibition leads to reduced fibroblast activation, blockade of collagen deposition, modulation of the fibrotic immune microenvironment [77].

Zelasudil (RXC007) acts as a selective inhibitor of Rho-Associated Coiled-Coil Containing Protein Kinase 2 (ROCK2).

A total of 48 patients with a confirmed diagnosis of IPF were enrolled in a 12-week phase 2a RCT NCT05570058 [78] to receive Zelasudil or placebo, administered twice daily (BID) at two exploratory doses: 20 mg BID and 50 mg BID. Patients could be on stable treatment with standard antifibrotic therapies (Nintedanib or Pirfenidone) or not take antifibrotics. The safety and tolerability profile was favourable: no treatment-related deaths or serious adverse events (SAEs) attributed to the drug were observed. The most frequent complication was asymptomatic elevations of transaminases, which resolved with treatment interruption or reduction. Regarding lung function, at the 20 mg BID dose the reduction in FVC decline compared to placebo was estimated at approximately 47% (−58 mL) after 12 weeks; at the 50 mg BID dose, the reduction was approximately 13% (−16 mL). In the extension study up to 24 weeks, patients who continued on Zelasudil demonstrated stabilization of lung function, and those who switched from placebo to Zelasudil also showed a trend toward stabilization [79]. The unexpected dose–response pattern may result from a bell-shaped pharmacological effect, where moderate ROCK2 inhibition yields optimal efficacy, while higher doses trigger compensatory mechanisms, off-target effects, or pharmacokinetic limitations that reduce therapeutic benefit.

However, the short duration (12–24 weeks) and limited number of patients do not allow for robust conclusions regarding long-term efficacy, exacerbations, survival, or quality of life. Larger studies over longer periods are needed for confirmation.

## 8. TNIK Pathway Inhibition

Tumor necrosis factor receptor–associated factor 2 and Nck-interacting kinase (TNIK) is a serine/threonine kinase of the germinal center kinase (GCK) family. Human TNIK is a 1360–amino acid protein composed of an N-terminal kinase domain, an intermediate domain, and a C-terminal citron homology (CNH) domain, which is conserved across the GCK family [80]. The intermediate domain mediates interactions with adaptor proteins such as TRAF2 and NCK, while the CNH domain participates in downstream signaling and cytoskeletal regulation. Through its kinase and CNH domains, TNIK modulates several intracellular pathways, including activation of the c-Jun N-terminal kinase (JNK) pathway, regulation of nuclear factor-κB (NF-κB) signaling, and control of actin cytoskeleton dynamics via interaction with the small GTPase Rap2 [80,81]. TNIK also plays a central role in Wnt/β-catenin signaling, acting as an essential activator by directly binding T-cell factor 4 (TCF4) and β-catenin through its kinase and intermediate domains, respectively [82]. Beyond Wnt signaling, TNIK has been implicated in additional pathways relevant to fibrotic and proliferative processes, including transforming growth factor-β (TGF-β), Merlin and focal adhesion kinase (FAK) signaling, as well as the PI3K–AKT–mTOR axis [80,83]. Through its ability to integrate signaling related to cell proliferation, differentiation, cytoskeletal organization and ECM regulation, TNIK represents a biologically compelling therapeutic target in diseases characterized by aberrant tissue remodeling, including pulmonary fibrosis. Notably, both TNIK as a therapeutic target and Rentosertib (formerly ISM001-055) as a compound in IPF were identified using a generative artificial intelligence platform [84]. Inhibition of TNIK with Rentosertib has demonstrated a reduction in fibroblast activation, ECM deposition (type I collagen, fibronectin), and cellular senescence in preclinical models [82]. A Phase 2a RCT called GENESIS-IPF (NCT05938920) [85] aimed to verify whether this treatment was safe and could offer benefits to patients with IPF. Patients were randomized to 12 weeks of treatment with different doses of Rentosertib (60 mg QD, 30 mg BID, 30 mg QD) or placebo. The primary endpoint was the percentage of patients with at least one treatment-emergent adverse event, which was similar across all treatment arms. Secondary endpoints included pharmacokinetic dynamics, changes in lung function as measured by FVC, DLCO and FEV1, change in the Leicester Cough Questionnaire score, change in 6 min walk distance and the number and hospitalization duration of acute exacerbations of IPF. An increase in FVC was observed in patients receiving the drug at the highest dosage, with a mean change of +98.4 mL (95% confidence interval 10.9 to 185.9) for patients in the 60 mg Rentosertib QD group, compared with −20.3 mL (95% confidence interval −116.1 to 75.6) for the placebo group [85]. Overall, these results indicate that TNIK inhibition with Rentosertib is safe and well tolerated, justifying further investigation in larger and longer-term clinical studies. Another phase IIa study in patients with IPF is currently ongoing [86].

## 9. Hedgehog (Hh) Signaling Inhibition

The Hedgehog (Hh) signaling pathway plays a fundamental role in controlling cell specification, proliferation, survival and tissue patterning formation during embryonic development [87].

In the adult lung, Hh signaling is largely quiescent and tightly regulated, contributing primarily to tissue homeostasis and repair following acute injury [88]. Under physiological conditions, low-level Hh activity supports epithelial integrity. Canonical Hh signaling is initiated by the binding of Hedgehog ligands, most notably Sonic Hedgehog (SHH), to the transmembrane receptor Patched-1 (PTCH1) [89]. In the absence of ligand, PTCH1 suppresses the activity of Smoothened (SMO), thereby preventing downstream signal transduction. Ligand binding relieves this inhibition, allowing SMO to activate the GLI family of transcription factors (GLI1, GLI2, and GLI3), which translocate to the nucleus and regulate gene expression programs involved in cell proliferation, survival, differentiation, and matrix regulation [87,90]. In pulmonary fibrosis, Hh signaling becomes aberrantly reactivated and dysregulated [91]. Injured and stressed alveolar epithelial cells, particularly AEC2, have been shown to upregulate SHH expression, leading to sustained paracrine activation of Hh signaling in adjacent mesenchymal cells. This persistent activation promotes fibroblast proliferation, myofibroblast differentiation, resistance to apoptosis, and enhanced production of ECM components such as collagen and fibronectin [92]. At the transcriptional level, increased GLI activity drives profibrotic gene expression and reinforces fibroblast persistence within fibrotic foci. As a result, Hh pathway reactivation contributes to fibrotic remodeling in IPF [87].

Targeting key components of this pathway has therefore emerged as a rational therapeutic strategy to disrupt fibroblast activation and attenuate fibrotic progression, providing the biological basis for the investigation of Hedgehog pathway inhibitors such as Taladegib.

Taladegib (ENV-101) is a selective oral inhibitor of Smoothened, a transmembrane protein in the Hh signaling pathway. This drug was evaluated in a phase 2a, 12-week RCT, NCT04968574 [93]. A total of 41 patients were randomized in a 1:1 ratio to receive 200 mg of Taladegib or placebo once daily. The primary endpoints were safety and tolerability and secondary endpoints included change from baseline to week 12 in absolute FVC, FVC percent predicted, and Quantitative Lung Fibrosis (QLF) by high resolution computed tomography (HRCT). Results demonstrated an improvement in percent predicted FVC of 1.9% in the drug arm versus a decline of 1.3% in the placebo group, along with a decrease in QLF by −9.4% in the drug arm versus an increase of 1.1% in the placebo arm. The most common TEAEs were dysgeusia, alopecia and muscle spasm, and five patients in the ENV-101 group discontinued the treatment due to drug-related side effects. The WHISTLE-PF trial (NCT06422884) is a phase 2b, 6-month, randomized, double-blind, controlled, dose-ranging study of Taladegib in IPF patients, currently recruiting [94].

Table 2 provides an overview of the most relevant therapies currently under investigation presented in the review.

## 10. Other Experimental Mechanisms and Targets Under Investigation in Pulmonary Fibrosis

This overview is intended to provide a broader perspective on the rapidly expanding therapeutic landscape in pulmonary fibrosis. Additional signaling pathways and candidate therapeutic molecules currently under investigation, including approaches supported primarily by experimental and preclinical evidence that could not be discussed in detail within the main text, are summarized in Table 3.

### 10.1. Integrins and ECM

Integrins play a pivotal role in the pathogenesis of pulmonary fibrosis through their involvement in the activation of TGF-β. In its inactive form, TGF-β is bound to latency-associated peptide and sequestered within the extracellular matrix [95]. Specific integrins, including αvβ6 expressed on injured AECs and αvβ1 expressed on fibroblasts, mediate the release and activation of TGF-β, thereby promoting fibroblast activation, myofibroblast differentiation, and collagen deposition [95]. Notably, αvβ6 expression is increased in IPF lung tissue, localizes to epithelial cells overlying fibrotic areas, and has been associated with disease severity and increased mortality [96]. Based on this mechanistic rationale, integrin inhibition with Bexotegrast, a dual small-molecule inhibitor of αvβ6 and αvβ1, emerged as a promising antifibrotic strategy. Early clinical evaluation in the phase IIa INTEGRIS-IPF trial suggested a favorable safety and tolerability profile, with a reduction in FVC decline over 12 weeks compared with those who received placebo, with or without background therapy [97]. However, subsequent longer-term evaluation in the phase IIb/III BEACON-IPF trial, NCT06097260 [98], revealed an unfavorable risk–benefit profile at higher doses, with increased rates of disease progression–related adverse events, leading to early termination of the study [99].

Excessive collagen production and deposition represent a central pathological feature of pulmonary fibrosis, making components of collagen biosynthesis and processing attractive therapeutic targets.

Prolyl-tRNA synthetase (PARS1/PRS), which catalyzes the incorporation of proline during collagen translation, has emerged as a regulator of collagen synthesis. Inhibition of PRS has demonstrated antifibrotic effects in bleomycin-induced pulmonary fibrosis models, leading to the clinical development of Bersiposocin (DWN12088), a PARS1 inhibitor currently under evaluation in a phase II randomized trial in patients with IPF (NCT05389215) [100].

An alternative strategy focuses on collagen maturation rather than synthesis. Heat shock protein 47 (HSP47) is a collagen-specific molecular chaperone essential for proper folding of procollagen within the endoplasmic reticulum and also participates in collagen turnover through interactions with matrix metalloproteinases and integrin signaling. Preclinical inhibition of HSP47 reduced fibrosis in experimental lung injury models; however, a phase II trial [101] of lipid nanoparticle–delivered siRNA targeting HSP47 (ND-L02-s0201) demonstrated an acceptable safety profile but failed to show efficacy in patients with IPF [102].

Additional experimental strategies focus on regulating ECM remodeling. Matrix metalloproteinase-7 (MMP7) is overexpressed in IPF, particularly in aberrant basaloid epithelial cells. Preclinical studies have shown that intratracheal silencing of MMP7 attenuates bleomycin-induced fibrosis and improves lung function [103], findings that were confirmed in non-human primates and human precision-cut lung slices using inhaled siRNA of MMP7 (ARO-MMP7). This approach is currently under evaluation in an ongoing phase I/II clinical trial in IPF, with results pending (NCT05537025) [104].

### 10.2. Thromboxane–Prostanoid Receptor Signaling

Thromboxanes are bioactive metabolites of arachidonic acid, functionally counterbalancing prostacyclin signaling, and have been implicated in the pathogenesis of idiopathic pulmonary fibrosis [105]. An increased thromboxane-to-prostacyclin ratio has been observed in IPF fibroblasts, suggesting a shift toward a profibrotic and vasoconstrictive milieu [106]. Consistently, the thromboxane–prostanoid receptor (TBXA2R) is upregulated in IPF lungs, particularly in fibroblasts, endothelial cells, and smooth muscle cells [106]. Preclinical studies have shown that genetic deletion or pharmacological inhibition of TBXA2R attenuates fibrotic remodeling, reducing profibrotic signaling and fibrosis severity across multiple experimental models, including bleomycin-, genetic-, and radiation-induced lung fibrosis [106]. On this basis, TBXA2R antagonism has emerged as a promising antifibrotic strategy, with the small-molecule Ifetroban currently under evaluation in a phase II randomized controlled trial in patients with IPF (NCT05571059) [107].

### 10.3. Oncostatin M–OSMRβ Signaling

Oncostatin M (OSM) is a pleiotropic cytokine of the IL-6 family that exerts both pro-inflammatory and profibrotic effects. OSM signaling through its receptor, OSMR-β, activates multiple downstream pathways, contributing to fibroblast proliferation, resistance to apoptosis, and increased ECM production [108,109]. Based on this mechanistic rationale, inhibition of OSMR-β has emerged as a potential antifibrotic strategy.

Vixarelimab, a fully human monoclonal antibody targeting OSMR-β, is currently under evaluation in a phase II randomized, placebo-controlled trial in patients with IPF and systemic sclerosis–associated ILD (NCT05785624) [110].

### 10.4. Janus Kinase (JAK)–STAT Signaling

The Janus kinase–signal transducer and activator of transcription (JAK–STAT) pathway plays a central role in immune regulation and inflammation. JAK family kinases (JAK1, JAK2, JAK3, and TYK2) phosphorylate STAT proteins, which subsequently translocate to the nucleus and regulate the transcription. Dysregulated activation of the JAK–STAT pathway has been implicated in a variety of inflammatory and autoimmune diseases, as well as in fibrotic lung disorders [111]. JAK inhibitors are small-molecule agents that suppress cytokine-driven signaling by blocking JAK enzymatic activity and are widely used in the treatment of hematologic malignancies, rheumatoid arthritis, and inflammatory skin diseases [112]. More recently, inhibition of the JAK–STAT pathway has gained attention in the context of ILDs [113]. A phase II trial investigating TTI-101 in IPF is currently recruiting (NCT05671835) [114]. In addition, JAK inhibitors, most notably Baricitinib, have demonstrated clinical benefit in severe COVID-19, reducing the need for mechanical ventilation and improving short-term survival [115]. On this basis, JAK inhibition has been explored as a potential therapeutic strategy for acute exacerbations of ILD [116].

### 10.5. Stem Cell Therapy

Stem cell–based therapies have attracted increasing interest as a potential regenerative strategy for pulmonary fibrosis. Different stem cell sources have been explored, including bone marrow, umbilical cord, adipose tissue, embryonic, induced pluripotent and endogenous lung stem/progenitor cells. Among these, mesenchymal stem cells (MSCs) derived from umbilical cord and bone marrow are the most extensively studied, largely due to their accessibility. Preclinical models have consistently shown that MSC administration reduces collagen deposition, attenuates fibroblast and myofibroblast activation, and suppresses profibrotic cytokines such as TGF-β1 and TNF-α [117], mainly through the secretion of paracrine mediators including prostaglandin E2 and hepatocyte growth factor [118].

The phase I trial AETHER demonstrated stem cells were safe and feasible to use in IPF [119]. Other phase I clinical trials on stem cell therapy for IPF are currently ongoing, NCT05016817 [120] and NCT05468502 [121]. In parallel, lung spheroid cells (LSCs), which consist of endogenous lung progenitor and support cells expanded ex vivo from lung tissue biopsies, have emerged as a novel regenerative approach. HALT-IPF [122] is a phase I study investigating autologous LSCs in the treatment of IPF, currently ongoing (NCT04262167). REGEND-001 represents a novel regenerative approach based on autologous airway repair, using patients’ own bronchial basal cells expanded ex vivo and re-administered to promote epithelial restoration in IPF. This strategy is now into phase II clinical evaluation (NCT06081621) [123]. Nebulized human umbilical cord MSC-derived extracellular vesicles demonstrated lung-targeted antifibrotic effects in preclinical models and were safe and associated with early functional improvement in a phase I clinical trial [124]. An early-phase clinical trial to assess the safety and efficacy of MSC therapy in PPF is currently ongoing NCT02594839 [125].

### 10.6. Multi-Target and Network-Based Therapeutic Approaches

Experimental evidence supports the antifibrotic potential of multi-component formulations acting on inflammatory and fibrogenic pathways through pleiotropic mechanisms. Using network pharmacology and molecular docking approaches, Astragalus-derived polysaccharides, a key component of several traditional Chinese formulations, have been identified as potential modulators of pulmonary fibrosis–related pathways [126]. This analysis revealed multiple putative targets involved in inflammation, oxidative stress, apoptosis, and fibrogenic signaling, including RELA (NF-κB), JUN, NOS2, SOD1, CASP3, and VCAM1, supporting a multi-pathway mechanism of action [126].

Extending this concept to multi-herbal formulations, network pharmacology and in vivo validation studies have investigated Buyanghuanwu Decoction (BYHWD), a classical Chinese medicine containing Astragalus among other components [127]. These analyses identified multiple active components targeting pathways involved in inflammation and oxidative stress. In bleomycin-induced pulmonary fibrosis models, BYHWD reduced fibrotic changes and downregulated key inflammatory mediators, including TNF, IL-6, IL-1β, and NOS2, while inhibiting NF-κB and p38 MAPK activation [127]. Although these findings remain preclinical, they reinforce the central role of inflammation-driven and NF-κB–dependent mechanisms in fibrogenesis and highlight the potential relevance of multi-target therapeutic strategies in pulmonary fibrosis.

### 10.7. Smad Signaling

Recent mechanistic studies have further refined the understanding of TGF-β/Smad regulation in pulmonary fibrosis by identifying transcriptional repressors that modulate Smad activity at the chromatin level [128]. FOXN3, a transcriptional repressor involved in inflammatory regulation, has been shown to exert antifibrotic effects by suppressing Smad-dependent transcription [129]. FOXN3 promotes Smad4 ubiquitination, thereby disrupting the Smad2/3/4 complex binding to target gene promoters and attenuating profibrotic gene expression [129]. In fibrotic conditions, this regulatory brake is lost through NEK6-mediated phosphorylation and subsequent degradation of FOXN3, resulting in stabilization of Smad chromatin binding and enhanced fibrogenic transcriptional activity. The inverse expression pattern of FOXN3 and Smad4 observed in human pulmonary fibrosis highlights the NEK6–FOXN3–Smad axis as a critical regulatory checkpoint controlling Smad-dependent transcriptional activity, rather than a simple upstream activator of TGF-β signaling [129]. This mechanism underscores how disruption of transcriptional repression can sustain profibrotic gene expression and contribute to disease progression.

**Table 3:** Novel molecular pathways and selected targeted therapies under investigation for Pulmonary Fibrosis.

Mechanism of Action	Drug/Agent	Clinical Trial	Development Status	References
Inhibition of CSF-1R signaling	Axatilimab	NCT06132256	Phase II	[130]
Inhibition of VEGFR, PDGFR, FGFR, and other kinases	Anlotinib	NCT05828953	Phase II/III	[131]
Antagonism of TBXA2R	Ifetroban	NCT05571059	Phase II	[107]
Inhibition of Autotaxin	BBT-877	NCT05483907	Phase II	[132]
	HNC1058	NCT05803850	Phase I	[133]
Inhibition of αvβ6 and αvβ1 integrins	Bexotegrast	NCT06097260	Phase IIb/III	[98]
MMP7 levels reduction	ARO-MMP7	NCT05537025	Phase I/IIa	[104]
Oncostatin M–OSMRβ signaling	Vixarelimab	NCT05785624	Phase II	[110]
Prolyl-tRNA synthetase 1 (PARS1) inhibition	Bersiposocin	NCT05389215	Phase II	[100]
C-Jun N-terminal kinase (JNK) inhibition	CC-9001	NCT03142191	Phase II	[134,135]
TGF-β1 mRNA reduction with siRNA	TRK250	NCT03727802	Phase I	[136,137]
Inhibition of Factor XII activity	Garadacimab	NCT05130970	Phase II	[138]
Inhibition of STAT3	TTI-101	NCT05671835	Phase II	[114]
Connective tissue growth factor (CTGF) inhibition	SHR-1906	NCT05722964	Phase II	[139]
TNF-α inhibition	Leramistat	NCT04312594	Phase II	[140]
Transglutaminase-2 (TG2) enzyme block	Zampilimab	NCT05513950	Phase Ib	[141]
Reactive oxygen species modulation	Setanaxib	NCT03865927	Phase II	[142]
Galectin-3 inhibition	GB0139	NCT03832946	Phase II	[143]
Heat shock protein 47 (HSP47) inhibition with siRNA	ND L02-s0201	NCT03538301	Phase II	[101]
Smad ubiquitination regulatory factor (SMURF) 1 inhibition	LTP-001	NCT05497284	Phase II	[144]
Tissue regeneration	Stem Cell Therapy	NCT05016817	Phase I	[120]
		NCT05468502	Phase I	[121]
		NCT04262167	Phase I	[122]
		NCT06081621	Phase II	[123]
		NCT06230822	Phase I	[145]

αvβ1, integrin alpha-v beta-1; αvβ6, integrin alpha-v beta-6; CSF-1R, colony-stimulating factor-1 receptor; CTGF, connective tissue growth factor; FGFR, Fibroblast Growth Factor Receptor; HSP47, heat shock protein-47; JNK, c-Jun N-terminal kinase; MMP7, matrix metalloproteinase-7; OSMRβ, oncostatin M receptor beta; PARS1, prolyl-tRNA synthetase-1; PDGFR, Platelet-Derived Growth Factor Receptor; ROS, reactive oxygen species; siRNA, small interfering RNA; SMURF1, Smad ubiquitination regulatory factor-1; STAT3, signal transducer and activator of transcription-3; TBXA2R, thromboxane-prostanoid receptor; TGF-β1, transforming growth factor beta-1; TG2, transglutaminase-2; TNF-α, tumor necrosis factor alpha; VEGFR, Vascular Endothelial Growth Factor Receptor. All clinical trials are identified by their ClinicalTrials.gov registration number (NCT).

## 11. Discussion

Despite remarkable progress in understanding the pathobiology of IPF and PPF, at the moment effective therapeutic options remain limited. Pirfenidone and Nintedanib have changed the therapeutic landscape by slowing disease progression, yet they are not able to halt or reverse fibrosis, and many patients experience treatment-limiting side effects.

The recent FDA approval of Nerandomilast, the first new drug for IPF in over a decade, represents a pivotal advance, offering a new therapeutic option to clinicians and renewed hope for patients.

It is expected that Nerandomilast can be used either as monotherapy in IPF (as first-line therapy or as a switch option for patients that do not tolerate the current medication) or as an add-on to Nintedanib or Pirfenidone in a combination therapy in patients with a progressive phenotype. In fact, given the complex pathophysiology of PPF, characterized by the interplay of inflammatory and fibrotic mechanisms, the multitargeted action of Nerandomilast positions it as a promising candidate for combination therapy with currently approved antifibrotic agents, as it happening in other respiratory and autoimmune diseases [146,147,148,149].

Future clinical development of emerging antifibrotic agents is expected increasingly to be based on add-on trial designs, in which these new therapies are combined with treatments of established efficacy, such as nintedanib or pirfenidone, a strategy likely to translate into combination regimens in routine clinical practice [150]. A recent systematic review [151] of seven studies including 238 patients receiving nintedanib (200–300 mg/day) plus pirfenidone (600–2400 mg/day) reported that 79% of patients experienced adverse events (AEs), with diarrhea, nausea, and vomiting being the most common, while only 6% experienced serious AEs and 25% discontinued therapy due to AEs. When compared with monotherapy, the overall AE rate was similar (80% vs. 79%), although discontinuations due to AEs were higher in combination therapy (34% vs. 10%, *p* = 0.001), and serious AEs were comparable (5% vs. 8%). Exploratory efficacy analyses suggested a trend toward slowing the decline in FVC, reaching statistical significance in two of five studies. The PROGRESSION trial (NCT03939520) is a currently ongoing Phase 4 study evaluating the efficacy and tolerance of the combination pirfenidone and nintedanib as compared to a switch monotherapy [152]. In this context, recent clinical evidence further supports the feasibility and potential benefit of combination therapy. For example, a multicenter, single-arm phase II study in patients with progressive fibrosing ILD [153] showed improved FVC trajectories with upfront immunomodulation (tacrolimus and low-dose prednisolone) combined with nintedanib, with manageable toxicity, supporting the feasibility of early combination strategies in PPF. Preclinical data indicate that nintedanib combined with pexidartinib, a colony-stimulating factor 1 receptor (CSF1R) inhibitor, effectively inhibited pulmonary fibrosis progression, promoted tissue repair, and modulated macrophage polarization in mouse models of bleomycin- and radiation-induced fibrosis [154].

Nonetheless, the road to combination therapy in IPF is likely to present multiple challenges. Tolerability is a key concern, as additive toxicity, particularly gastrointestinal side effects, is common. Evaluating efficacy is also complex, as trial endpoints must balance short-term prediction with long-term trajectory of disease [155]. Traditional single endpoints, such as FVC decline or mortality, may not fully reflect the multifaceted nature of IPF and other progressive fibrosing ILDs. Composite endpoints, which integrate multiple measures, such as FVC change, DLCO, symptom scores, imaging findings, and clinical events, might provide a more comprehensive assessment of disease trajectory, offering a practical framework to better evaluate the effectiveness of novel combination regimens while accounting for the heterogeneous course of disease [156,157].

Thanks to the identification of novel pathogenic pathways and the development of targeted molecules directed against them [158,159,160], the therapeutic horizon is expanding, paving the way for a new generation of antifibrotic drugs aimed at reducing disease burden, acute exacerbations and its associated mortality [29], and improving outcomes.

The shift toward a phenotype-based framework in interstitial lung diseases, centered on progressive disease behavior rather than underlying etiology, has important implications for emerging antifibrotic therapies. Several novel pathways discussed in this review target core fibrotic mechanisms. At the moment these pathways may be considered largely phenotype-agnostic and conceptually aligned with current PPF treatment strategies. In fact, the lack of biomarkers capable of predicting treatment response, a problem already encountered with currently approved therapies, similarly affects emerging targeted agents, for which pathway-specific predictive or response-associated biomarkers remain largely unexplored or unvalidated in clinical trials. This limits the implementation of truly personalized therapeutic approaches. Should reliable biomarkers be identified within these novel pathways, and corresponding therapies demonstrate clinical efficacy, this would represent a major advance, enabling more effective patient stratification, treatment monitoring, and the application of personalized therapeutic strategies. In the current absence of validated biomarkers capable of guiding treatment selection, the combined use of circulating biomarkers and high-resolution computed tomography (HRCT)–derived quantitative imaging features represents the most feasible approach to improve fibrosis phenotyping, disease monitoring, and therapeutic assessment [28,161,162]. Consequently, emerging antifibrotic agents are expected to be primarily developed and used in combination with approved therapies, rather than as standalone, phenotype-restricted treatments, reflecting the biological complexity of progressive fibrotic lung disease.

## 12. Conclusions

Although our understanding of the pathogenesis of pulmonary fibrosis has advanced considerably, IPF and PPF continue to represent conditions with a high unmet clinical need. Currently approved antifibrotic therapies slow disease progression but are not curative, and prognosis remains poor. In recent years, increasing insight into the molecular and cellular mechanisms underlying fibrogenesis has led to the identification of multiple novel therapeutic targets acting across epithelial injury, fibroblast activation, immune dysregulation and ECM dynamics. Several agents have shown encouraging signals in early- and mid-stage clinical development, and phase III programs—such as those evaluating treprostinil and admilparant—represent important milestones in this evolving landscape. At the same time, promising phase II data targeting pathways including ROCK2, TNIK, and angiotensin II type 2 receptor signaling further support the biological relevance of pathway-directed interventions.

Future progress will depend on the integration of robust preclinical models, carefully designed clinical trials with meaningful and patient-centered endpoints, and deeper mechanistic insight into disease pathogenesis, since a more refined understanding of pathways involved in the disease may ultimately enable the development of mechanism-based treatment strategies, a critical step toward earlier intervention and improved care for patients with pulmonary fibrosis.

## Figures and Tables

**Table 2:** Most Promising Emerging Therapies for Pulmonary Fibrosis.

Drug/Agent	Mechanism/Target Pathway	Clinical Trial	Main Findings	Development Status
Nerandomilast	PDE4B inhibition → ↑cAMP; anti-inflammatory and antifibrotic modulation	FIBRONEER-IPF & FIBRONEER-ILD (Phase III)	Significantly reduced FVC decline vs. placebo; good tolerability [39,40]	FDA approved (2025) for IPF; Ongoing FDA evaluation for approval also in PPF
Inhaled Pirfenidone (AP01)	Local delivery of antifibrotic pirfenidone; ↓fibroblast activation	Phase Ib (ACTRN12618001838202)	Stabilized FVC (–0.4% at 48 weeks, high-dose group); minimal systemic side effects [44]	Phase IIb ongoing
Pirfenidone analogues (Sufenidone/Yfenidone)	Structural analogues of pirfenidone; improved tolerability	Phase II/III (NCT06125327, NCT05060822, NCT05139719)	Under investigation	Phase II–III ongoing
Admilparant (BMS-986278)	LPA_1_ receptor antagonist → blocks LPA/ATX fibrotic signaling	Phase II (NCT04308681)	Slowed FVC decline; favourable safety profile [55]	Phase III NCT06003426 for IPF and NCT06025578 for PPF ongoing
Treprostinil	Prostacyclin analogue → ↑cAMP, inhibits TGF-β signaling	INCREASE (Phase III)	Improved exercise tolerance, stabilized FVC decline [63]	Phase III TETON-IPF/TETON-PPF ongoing
Buloxibutid (C21)	AT_2_ receptor agonist → epithelial repair, antifibrotic	AIR (Phase IIa, NCT04533022)	Stable or improved FVC; reduced TGF-β1, increased MMP-13 [72]	Phase IIb ASPIRE NCT06588686 ongoing
Zelasudil (RXC007)	ROCK2 inhibition → ↓ fibroblast activation, ECM deposition	Phase IIa (NCT05570058)	47% reduction in FVC decline at 12 weeks; favourable safety profile [79]	Phase IIa completed; further studies planned
Rentosertib (ISM001-055)	TNIK inhibition → blocks Wnt/β-catenin and TGF-β signaling	GENESIS-IPF (Phase IIa, NCT05938920)	Increased FVC (+98 mL) at 12 weeks; well tolerated [85]	Phase IIa completed; further studies planned
Taladegib (ENV-101)	Inhibitor of Smoothened, involved in hedgehog signaling pathway	Phase IIa (NCT04968574)	Increased FVC, decrease in QLF at 12 weeks [93]	Phase IIb ongoing

## Data Availability

No new data were created or analyzed in this study.
